# Quantitative phosphoproteomics analyses reveal the regulatory mechanisms related to frozen-thawed sperm capacitation and acrosome reaction in yak (*Bos grunniens*)

**DOI:** 10.3389/fphys.2022.1013082

**Published:** 2022-10-06

**Authors:** Renzheng Zhang, Chunnian Liang, Xian Guo, Pengjia Bao, Jie Pei, Fude Wu, Mancai Yin, Min Chu, Ping Yan

**Affiliations:** ^1^ Key Laboratory of Animal Genetics and Breeding on Tibetan Plateau, Ministry of Agriculture and Rural Affairs, Key Laboratory of Yak Breeding Engineering of Gansu Province, Lanzhou Institute of Husbandry and Pharmaceutical Sciences, Chinese Academy of Agricultural Sciences, Lanzhou, China; ^2^ College of Animal Science and Technology, Gansu Agricultural University, Lanzhou, China; ^3^ Yak Breeding and Extension Service Center in in Qinghai Province, Xining, China

**Keywords:** yak sperm, capacitation, acrosome reaction, post-translational modifications, phosphorylation

## Abstract

Mammalian spermatozoa are not mature after ejaculation and must undergo additional functional and structural changes within female reproductive tracts to achieve subsequent fertilization, including both capacitation and acrosome reaction (AR), which are dominated by post-translational modifications (PTMs), especially phosphorylation. However, the mechanism of protein phosphorylation during frozen-thawed sperm capacitation and AR has not been well studied. In this study, the phosphoproteomics approach was employed based on tandem mass tag (TMT) labeling combined with liquid chromatography-tandem mass spectrometry (LC-MS/MS) strategy to analyze frozen-thawed sperm in Ashidan yak under three sequential conditions (density gradient centrifugation-based purification, incubation in the capacitation medium and induction of AR processes by the calcium ionophore A23187 treatment). The identification of 1,377 proteins with 5,509 phosphorylation sites revealed changes in phosphorylation levels of sperm-specific proteins involved in regulation of spermatogenesis, sperm motility, energy metabolism, cilium movement, capacitation and AR. Some phosphorylated proteins, such as AKAP3, AKAP4, SPA17, PDMD11, CABYR, PRKAR1A, and PRKAR2A were found to regulate yak sperm capacitation and AR though the cAMP/PKA signaling pathway cascades. Notably, the phosphorylation level of SPA17 at Y156 increased in capacitated sperm, suggesting that it is also a novel functional protein besides AKAPs during sperm capacitation. Furthermore, the results of this study suggested that the phosphorylation of PRKAR1A and PRKAR2A, and the dephosphorylation of CABYR both play key regulatory role in yak sperm AR process. Protein-protein interaction analysis revealed that differentially phosphorylated proteins (AKAP3, AKAP4, FSIP2, PSMD11, CABYR, and TPPP2) related to capacitation and AR process played a key role in protein kinase A binding, sperm motility, reproductive process, cytoskeleton and sperm flagella function. Taken together, these data provide not only a solid foundation for further exploring phosphoproteome of sperm in yak, but an efficient way to identify sperm fertility-related marker phosphorylated proteins.

## Introduction

Even morphologically normal and motile sperm fail to fertilize an oocyte immediately after ejaculation, and must instead stay in female genital tracts for an appropriate time to gain the ability to fertilize the egg through a process called capacitation (Austin, 1951; Chang, 1951). This involves both biochemical and biophysical changes, including the cholesterol efflux from the plasma membrane, leading to membrane fluidity and the increased permeability to bicarbonate and calcium ions, the plasma membrane hyperpolarization (Hernandez-Gonzalez et al., 2006) and changes in protein phosphorylation and protein kinase activity (Arcelay et al., 2008; Visconti, 2009). These changes jointly endow sperm with the ability to recognize and fertilize eggs, and they are also prerequisites for AR (Aitken and Nixon, 2013; Castillo et al., 2019; Peris-Frau et al., 2019).

Mature sperm are highly differentiated and specialized cells. Therefore, capacitation is remarkably characterized by its occurrence in the complete absence of nuclear gene transcription and *de novo* protein synthesis. On the contrary, the regulation of capacitation depends almost entirely on convergent signaling cascades, which transduce extracellular signals to achieve a wide range of PTMs of the sperm intrinsic proteome (reviewed by Gervasi and Visconti, 2016). Studies confirmed that phosphorylated proteins, protein kinases and phosphatases play an important role in the recognition and adhesion of sperm motility, capacitation and sperm-oocyte, the ability to undergo acrosome exocytosis and hyperactivated motility in mammalian sperm (Garbers et al., 1980; Tash and Means, 1983; Stival et al., 2016). In this context, the protein regulation at the PTM level, especially phosphorylation, has emerged as a dominant molecular switch regulating these functions. As a matter of fact, it is generally believed that increased levels of tyrosine phosphorylation mediated by activating PKA activity through increased intracellular cAMP levels are critical for sperm capacitation (Battistone et al., 2013; Martinez-Leon et al., 2015). In turn, intracellular cAMP can dissociate the regulatory subunits of protein kinase A (PKAr) from its corresponding catalytic subunits (PKAc), with the release of PKAc in an activated state, and also promote a significant increase in serine/threonine-based phosphorylation in capacitated sperm (Visconti et al., 1995; Nolan et al., 2004; Baker et al., 2006). This fully demonstrated that serine/threonine also plays a crucial role during sperm capacitation. However, the phosphorylation research on sperm capacitation has mainly focused on tyrosine so far, with few reports on serine/threonine (Chung et al., 2014; Wang et al., 2015; Syifa et al., 2020). All these changes occurred before the AR, which is a Ca^2+^ dependent process (Buffone et al., 2008), including the activation of phospholipases C and A2 as well as PKC and cAMP-dependent protein kinase pathways, implying that protein phosphorylation plays a major role in the secondary messenger regulatory mechanism of AR (Benoff, 1998; Topfer-Petersen et al., 2000; Urner and Sakkas, 2003; Ashizawa et al., 2006). If phosphorylation is required to activate the AR under the active condition of cAMP-triggered events, the acceleration of the serine/threonine dephosphorylation of some phosphorylated proteins by phosphatase will be indispensable for AR (Ashizawa et al., 2004; Mizuno et al., 2015; Ruete et al., 2019). However, the regulatory mechanism of protein phosphorylation in sperm AR has not been studied. The calcium ionophore A23187 induced sperm AR is mainly due to the opening of calcium channels and thereby increase calcium influx, and calcium ionophore A23187 increases the cAMP and PKA levels in spermatozoa (Lefievre et al., 2002) and by-passes the initial signaling mechanism of the AR induced by the zona pellucida, which involves phosphorylation protein-regulated mechanisms (Breitbart and Spungin, 1997; Grasa et al., 2009).

Yak are livestock unique to the high-altitude area (2,500–5,500 m) of the Qinghai-Tibet Plateau, and they are in seasonal estrus, with a breeding period from July to October and a calving period from April to July. Female yak breed once every other year or twice every 3 years and have a reproductive rate 40%–60% lower than other livestock (Sarkar and Prakash, 2005; Pan et al., 2015). Semen cryopreservation is an effective method for preserving male fertility and introducing desired superior genetic traits in animal husbandry. However, cryopreservation can produce some side effects, including decreased sperm motility, excessive production of reactive oxygen species, lipid peroxidation, changes in the composition and permeability of the cell membrane, and impaired mitochondrial activity, thus resulting in ATP consumption (Pini et al., 2018; Grotter et al., 2019; Peris-Frau et al., 2020). Owing to changes in membrane fluidity and calcium influx, surviving sperm immediately undergo a capacitation-like process immediately upon thawing (Watson, 1995), requiring less time to achieve capacitation as compared to fresh sperm (Peris-Frau et al., 2020). As a matter of fact, the molecular mechanisms of regulating the capacitation process of fresh and frozen-thawed sperm are considered to be distinct (Peris-Frau et al., 2019; Peris-Frau et al., 2020). Frozen-thawed sperm are more commonly used for *in vitro* production of embryos and artificial insemination. In previous studies, the phosphorylation regulatory mechanism related to capacitation in fresh sperm of humans and mice without sublethal damage was explored (Platt et al., 2009; Baker et al., 2010; Chung et al., 2014; Wang et al., 2015; Syifa et al., 2020). The phosphorylated protein change caused by cryopreservation is an important factor affecting sperm motility and fertilization function (Wang et al., 2021). However, the regulatory mechanism of phosphorylated proteins related to the capacitation and AR induction of frozen-thawed sperm has not yet been determined.

In this study, the phosphopeptides were enriched by the immobilized metal affinity chromatography (IMAC) method and subjected to LC-MS/MS analysis with TMT labeling strategy to uncover PTMs involving sperm capacitation and AR in yak. The identified phosphorylated proteins have the potential to become an important mediator of sperm function and related fertility, which provides new insights into the mechanisms underlying the gradual maturation of sperm after ejaculation through combined phosphoproteomics and bioinformatic analysis. By further understanding the specific phosphorylated proteins as well as their sites and signal pathways that affect sperm capacitation and AR induction, this study will provide new insights into the molecular mechanism of improving reproductive efficiency in yak.

## Materials and methods

### Reagents

All chemicals were obtained from Sigma-Aldrich (St. Louis, MO, United States) unless otherwise indicated.

### Sample collection

**FIGURE 1:**
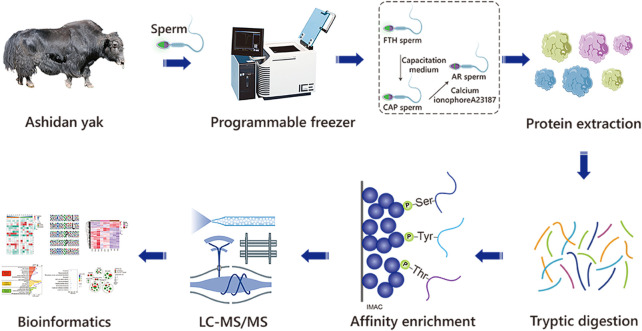
The workflow of the experimental procedures. The identification of phosphoproteomics in yak sperm was performed by TMT labeling combined with LC-MS/MS after capacitation and AR *in vitro*.

An artificial vagina was used to collect ejaculates from 3 Ashidan yaks (5.5 ± 0.62 years) from the yak breeding center in Qinghai Province, China. Ashidan yak are unique hornless breed of yak bred by the Lanzhou Institute of Husbandry and Pharmaceutical Sciences of the Chinese Academy of Agricultural Sciences. After collection, ejaculates were immediately assessed *via* computer-assisted sperm analysis (CASA, Minitube, GER) to assess progressive motility, with only those ejaculates with a volume higher than 2.0 ml and s exhibiting >70% progressive motility accepted for subsequent use. A two-step dilution method was adopted with Biladyl^®^ extender (Minitube, GER). First, the without glycerol fraction was added (50% of the calculated final volume), diluted and cooled at 5°C for 2 h. Then, the samples were further diluted by adding pre-cooled (5°C) glycerol-containing fractions and kept at 5°C for 2 h, and sperm samples were frozen to a final concentration of 140–200 × 10^6^/ml. Next, a cooling curve of −5°C/min was used from 5°C to 4°C, then −3°C/min was used from 4°C to −10°C, then −40°C/min was used from −10°C to −100°C, then −20°C/min was used from −100°C to −140°C, and placed in a liquid nitrogen storage tank. For use in subsequent experiments, these samples were processed under three sequential conditions (Figure 1), including frozen-thawed high motility sperm (FTH sperm), sperm incubated in the capacitation medium (CAP sperm), and sperm of AR induction by calcium ionophore A23187 treatment (AR sperm).

### Density-gradient centrifugation

Briefly, sperm-containing straws were thawed for 30 s at 37°C before being added to a prepared 45%–90% Percoll solution (Percoll P1644; 3.1 mM KCl, 2.9 mM NaH_2_PO_4_, 80 mM NaCl, 10 mM HEPES, 2 mM CaCl_2_·H2O, 0.4 mM MgCl_2_ 6H_2_O, 26 mM Sodium DL-Lactate Solution, 25 mM NaHCO_3_; pH 7.3–7.4), and diluted with modified Tyrode’s Hepes buffered medium (Sp-TALPH, 114 mM NaCl, 3.2 mM KCl, 2 mM NaHCO_3_, 0.4 mM NaH_2_PO_4_·H_2_O, 10 mM Na Lactate, 2 mM CaCl_2_·H_2_O, 0.5 mM MgCl_2_ 6H_2_O, 10 mM HEPES, 3 mg/ml BSA, 0.2 mM Na pyruvate, 7.5 × 10^−3^ mg/ml Gentamicin; pH 7.3–7.4) (Galantino-Homer et al., 1997; Thundathil et al., 2006), followed by centrifugation for 10 min at 700 xg to isolate highly motile sperm. Then, in order to ensure the complete elimination of gradient materials from these samples, the sperm cells collected from the bottom layer were washed two times with supplemented Sp-TALPH and centrifuged for 5 min at 300 xg. Next, sperm were isolated and adjusted to a concentration of 100 × 10^6^/ml by using modified Tyrode’s bicarbonate buffered medium (Sp-TALP; 114 mM NaCl, 3.2 mM KCl, 25 mM NaHCO_3_, 0.4 mM NaH_2_PO_4_·H_2_O, 10 mM Na Lactate, 2 mM CaCl_2_·H_2_O, 0.5 mM MgCl_2_ 6H_2_O, 6 mg/ml BSA, 0.2 mM Na pyruvate, 5 × 10^−3^ mg/ml Gentamicin; pH 7.3–7.4) (Parrish et al., 1988; Thundathil et al., 2006). After that, a CASA approach was adopted to assess sperm motility, with only the samples of >70% progressive motility retained for further analyses, according to previously published protocols (Zhang et al., 2022). Finally, an aliquot containing 100 million FTH sperm was taken from each sample for subsequent protein isolation and the remaining sperm were incubated in the capacitation medium.

### Sperm capacitation

The capacitation medium was composed of Sp-TALP containing heparin (50 µg/ml) and caffeine (5 mM), and sperm were incubated in it at 38.5°C for 30 min in a 5%CO_2_ incubator, which has previously been shown to be optimal for capacitation induction in frozen-thawed yak sperm (Zhang et al., 2022). An aliquot containing 100 million CAP sperm was extracted from each sample for subsequent protein isolation, with the remaining sperm treated with A23187.

### Acrosome reaction induction

AR induction was performed by treating 990 µl capacitated sperm samples with 10 µl A23187 (1.0 mM in DMSO, CSNpharm, IL, United States) to a final concentration of 10 µM, followed by incubation at 38.5°C for 15 min according to the World Health Organization (WHO) guidelines and a slightly modified version of previously published protocols (Castillo et al., 2019; Hou et al., 2019). For single replicates, aliquots of 100 million AR sperm were collected for subsequent protein analyses. For negative controls, sperm were treated with DMSO under identical conditions (De Jonge and Barratt, 2013; Castillo et al., 2019).

### Analyses of acrosome reaction status

The percentage of sperm from FTH, CAP, and AR sperm samples that had undergone AR was assessed based on sperm acrosome vesicle integrity measured with the PSA-FITC kit (GENMED, MA, United States). Briefly, 20 µl sperm samples was smeared onto a glass slide, allowed to air-dry, fixed for 1 min with methanol, and stained for 20 min by PSA-FITC kit under the protection from light. Then, samples were washed and AR status was examined by fluorescence microscopy (Olympus, BX51, Tokyo, Japan). The fluorescent probe was utilized to bind to alpha-methyl mannose, thereby labeling sperm acrosomal content. In total, 200 sperm per slide were assessed and counted by a technician blinded to sample grouping. Sperm were identified as showing either an intact acrosome so that over half of sperm heads exhibited bright green uniform fluorescence, or a reacted acrosome so that only an equatorial band of green fluorescence or none was evident in the acrosomal region.

### Protein extraction and trypsin treatment

First, FTH, CAP, and AR sperm samples were rinsed two times in PBS, followed by resuspension in a lysis buffer composed of 1% SDS supplemented with protease and phosphatase inhibitors. Second, these homogenates were subjected to three rounds of sonication by a high-intensity ultrasonic processor (Ningbo Scientz Biotechnology Co., Ltd., China). Third, samples were centrifuged at 12,000 xg at 4°C for 10 min, followed by the collection of supernatants, and the protein levels in them were quantified by BCA kit (Beyotime Biotechnology, Wuhan, China) according to the provided instructions.

The protein sample was added with 1 volume of pre-cooled acetone, vortexed to mix, and added with 4 volumes of pre-cooled acetone, precipitated at −20°C for 2 h. The protein sample was redissolved in 200 mM TEAB and ultrasonically dispersed, followed by the addition of Trypsin at a 1:50 mass ratio of trypsin-to-protein for the first digestion overnight. After that, the samples were reduced with 5 mM dithiothreitol at 37°C for 60 min and alkylated with 11 mM iodoacetamide for 45 min at room temperature in the dark. Finally, the peptides were desalted by the Strata X SPE column.

### Tandem mass tag labeling and HPLC fractionation

Digested peptides were desalted by a Strata X C18 SPE column (Phenomenes, United States), and then vacuum-dried, dissolved with 0.5 M TEAB and labeled with a TMT kit (Thermo, United States) according to provided instructions. Next, proteins were divided into fractions *via* high pH reverse-phase HPLC by using a Thermo Betasil C18 column (5 μm particles, 10 mm ID, 250 mm length). After that, peptides were initially separated into 60 fractions in 60 min by an 8%–32% acetonitrile (pH 9.0; Fisher Chemical, United States) gradient. And they were combined into 6 fractions and dried by vacuum centrifugation.

### Affinity enrichment of phosphopeptides

First, the peptide mixture was incubated along with IMAC microsphere suspension by vibration in loading buffer (50% acetonitrile/6% trifluoroacetic acid). Then, the IMAC microspheres containing enriched phosphopeptides were collected by centrifugation, with the removal of the supernatant. Next, in order to remove nonspecifically adsorbed peptides, the IMAC microspheres were washed sequentially with 50% acetonitrile/0.5% trifluoroacetic acid and 30% acetonitrile/0.1% trifluoroacetic acid. After that, the elution buffer containing 10% NH_4_OH was added to elute the enriched phosphopeptides from the IMAC microspheres by vibration. Finally, the eluted fractions were combined and vacuum-dried. The resulting peptides were desalted with C18 ZipTips (Millipore) for LC-MS/MS analyses according to the manufacturer’s instructions.

### Liquid chromatography-tandem mass spectrometry analyses

First, after tryptic peptides were all dissolved with 0.1% formic acid, they were directly loaded on a homemade reversed-phase analytical column. Second, the gradient elution was performed by increasing solvent B (0.1% formic acid in 98% acetonitrile) concentrations from 4 to 20% in 40 min, from 20 to 32% in 12 min, and from 32 to 80% in 4 min, respectively. Third, the samples were kept in 80% solvent B for 4 min. The flow rate was maintained at 450 nl/min by an EASY-nLC 1200 UPLC system (Thermo Fisher Scientific, CA, United States). Then, peptides were introduced into an NSI source, followed by MS/MS in a Q ExactiveTM HF-X (Thermo Fisher Scientific) coupled online to the UPLC apparatus. Next, the electrospray voltage was set to 2.2 kV, and peptides were detected in the Orbitrap at a resolution of 60,000, with a full scan range of 350–1,400 m/z. After that, a normalized collision energy (NCE) setting of 28 was used to detect selected MS/MS peptides at 30,000 resolution in the Orbitrap. A data-dependent procedure was alternated between one MS and 20 MS/MS scans with a 15.0 s dynamic exclusion. Finally, the automatic gain control (AGC) was set to 5E4, and the fixed first mass was set to 100 m/z.

### Database search

The Maxquant search engine (v.1.5.2.8) was used to analyze the resulting data by comparing MS/MS spectra with the Uniprot bovine database (Yang et al., 2020), concatenated with a reverse decoy database. Trypsin/P was specified as the utilized cleavage enzyme with up to 2 missing cleavages allowed. The mass ion tolerance was set to 20 ppm for the initial search, and limited to 5 ppm for the main search (Fragment ions: 0.02 Da). Carbamidomethyl on Cys was specified as a fixed modification, and Acetylation on the N-terminus of the protein, oxidation on Met, deamidation (NQ) and phosphorylation on Ser, Thr, and Tyr as a variable modification. TMT-10plex quantification was performed. The FDR was set to <1%, and the minimum peptide score was >40.

### Motif analyses

Software Momo and motif-x (http://motif-x.med.harvard.edu/motif-x.html) algorithm were adopted to analyze the motif characteristics of modification sites. The peptide fragment sequence composed of 6 amino acids upstream and downstream each of all identified modification sites acted as the analysis object (Chou and Schwartz, 2011). The protein sequences in the database were used as the background parameters, with other parameters set by default. When the number of peptide segments in the form of a characteristic sequence >20 and the statistical test *p*-value< 0.000001, the characteristic sequence form would be considered a motif of modified peptide segments.

### Bioinformatics analyses

BioMart was used to convert protein IDs to Ensembl gene IDs such that the data would be comparable with other datasets (Li Y. et al., 2020). Differentially expressed proteins were analyzed by the Gene Ontology (GO) annotations (http://www.geneontology.org/) and the Kyoto Encyclopedia of Genes and Genomes (KEGG) pathway (http://www.genemo.jp/kegg/). In bioinformatics analysis, *p* < 0.05 was the threshold for significant enrichment. Cello (version 2.5) was used for subcellular localization predication. STRING v11.5 (https://string-db.org/) was adopted to retrieve known protein-protein interaction (PPI), which were used to establish a PPI network by Cytoscape 3.9.0, with the network of ≤2 nodes excluded (Shannon et al., 2003).

## Results

### The confirmation of successful capacitation and acrosome reaction induction

**FIGURE 2:**
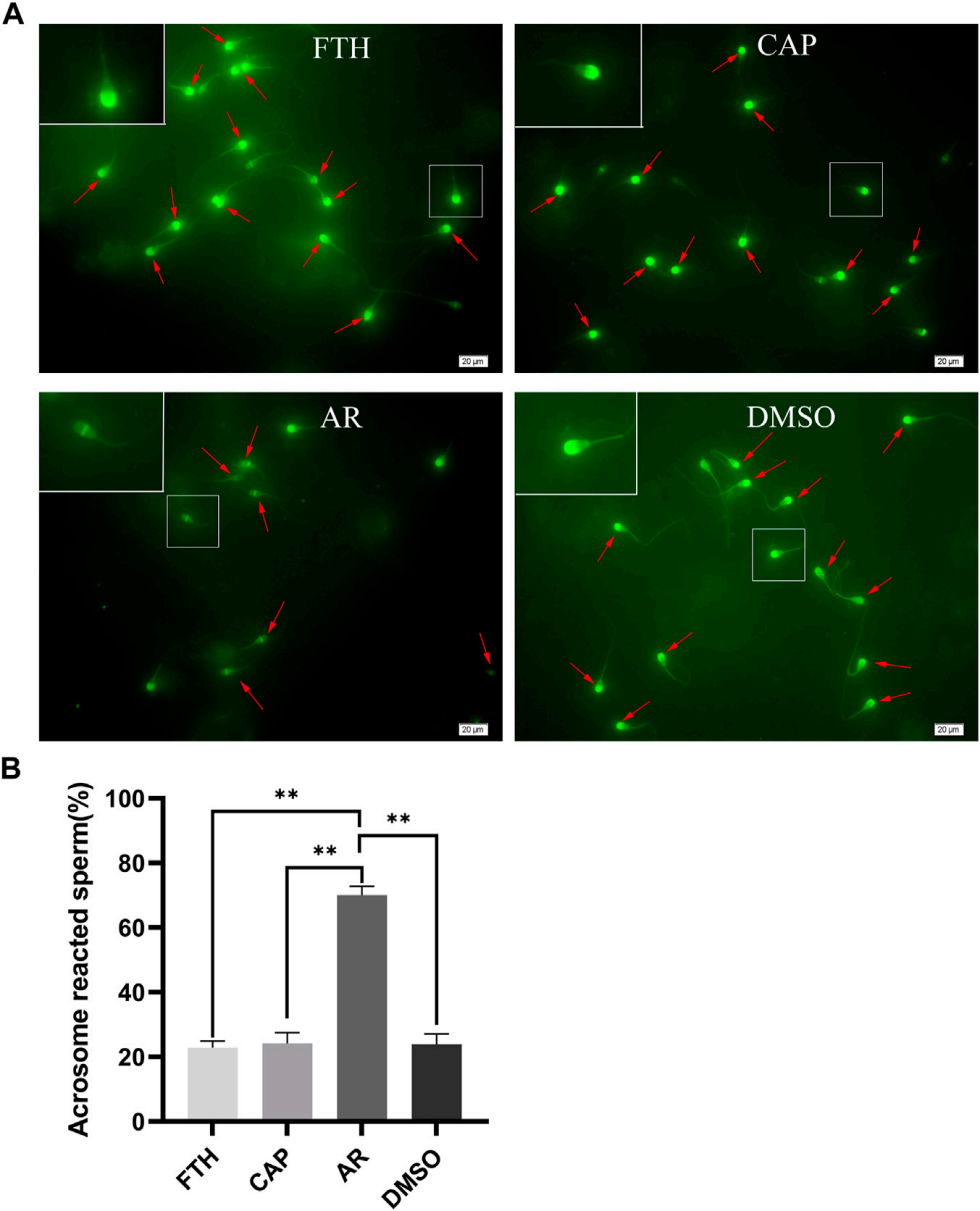
The analysis of acrosome integrity based on PSA-FITC Labeling. **(A)**Representative images of PSA-FITC-labeled frozen-thawed sperm in yak. Sperm were isolated by 45%–90% Percoll density gradient centrifugation (FTH, upper left), incubated in the capacitation medium containing 50 µg/ml heparin and 5 mM caffeine (CAP, upper right), and treated with the calcium ionophore A23187 to induce AR (AR, lower left). DMSO (lower right) served as a vehicle control. The inset white squares denote magnified sperm; arrows mark acrosomal caps; scale bar, 20 µm. **(B)** The quantification of the number of sperm with reacted acrosomes in the indicated samples after PSA-FITC labeling (**p < 0.01).

Before proteomic analyses, a PSA-FITC labeling strategy was performed to examine sperm acrosomal integrity in the FTH, CAP, and AR sperm samples as a means of indirectly assessing sperm capacitation (Figure 2A). In this analysis, sperm were found to exhibit either an intact or reacted acrosome. The percentage of sperm with reacted acrosomes was significantly increased in AR sperm samples, compared with the FTH or CAP sperm ones, thus indirectly confirming that these sperm have undergone efficient capacitation (Figure 2B). Importantly, DMSO treatment for the negative control had no impact on AR induction when AR sperm were compared with those in the FTH or CAP sperm samples (one-way ANOVA with Tukey’s multiple comparison test, *p* < 0.01).

### Identification and quantification of phosphopeptides in yak sperm

**FIGURE 3:**
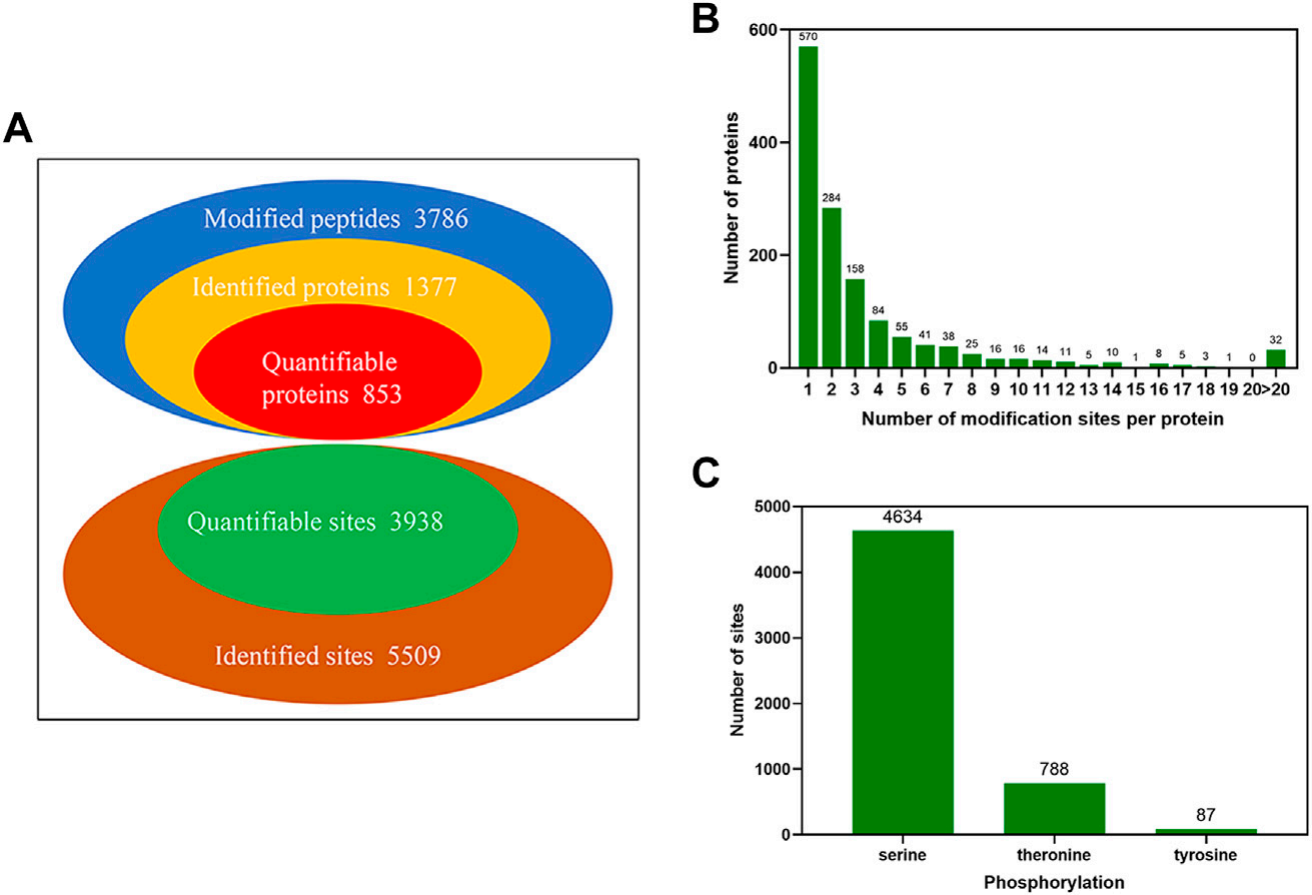
The identification of global phosphorylated proteins and their sites in yak sperm. **(A)** The identification and quantification of phosphorylated proteins and sites. **(B)** The number distribution of phosphorylation modification sites of phosphorylated proteins. **(C)** The phosphorylation site distribution of serine, threonine and tyrosine.

A TMT labeling combined with LC-MS/MS was quantitative strategy adopted to investigate the phosphorylated proteome in yak sperm. Under three sequential conditions of FTH, CAP, and AR sperm in yak, a total of 5,509 phosphorylation sites were identified, with 1,377 phosphorylated proteins matched. Among them, 3,938 sites of 853 proteins were quantified (Localization probability of >0.75; Andromeda score >40; PEP score >0.06 (Figure 3A; Supplementary Table S1). In order to evaluate the distribution of phosphorylation sites in yak sperm, statistical analysis of the number of each phosphorylated protein modification site identified was performed. The results showed that 570 proteins contained one phosphorylation site (41.4%), 284 proteins two phosphorylation sites (20.6%), and 158 proteins three phosphorylation sites (11.5%). In addition, there were more than 10 phosphorylation sites (6.5%) in 90 proteins, and there were more than 20 phosphorylation sites (2.3%) in 32 proteins (Figure 3B). Moreover, the distribution of serine, threonine and tyrosine at phosphorylation sites was calculated, of which 4,634 were located at serine (84.1%), 788 were located at threonine (14.3%), and 87 were located at tyrosine (1.5%) (Figure 3C).

### Motif analyses of phosphorylation sites

**FIGURE 4:**
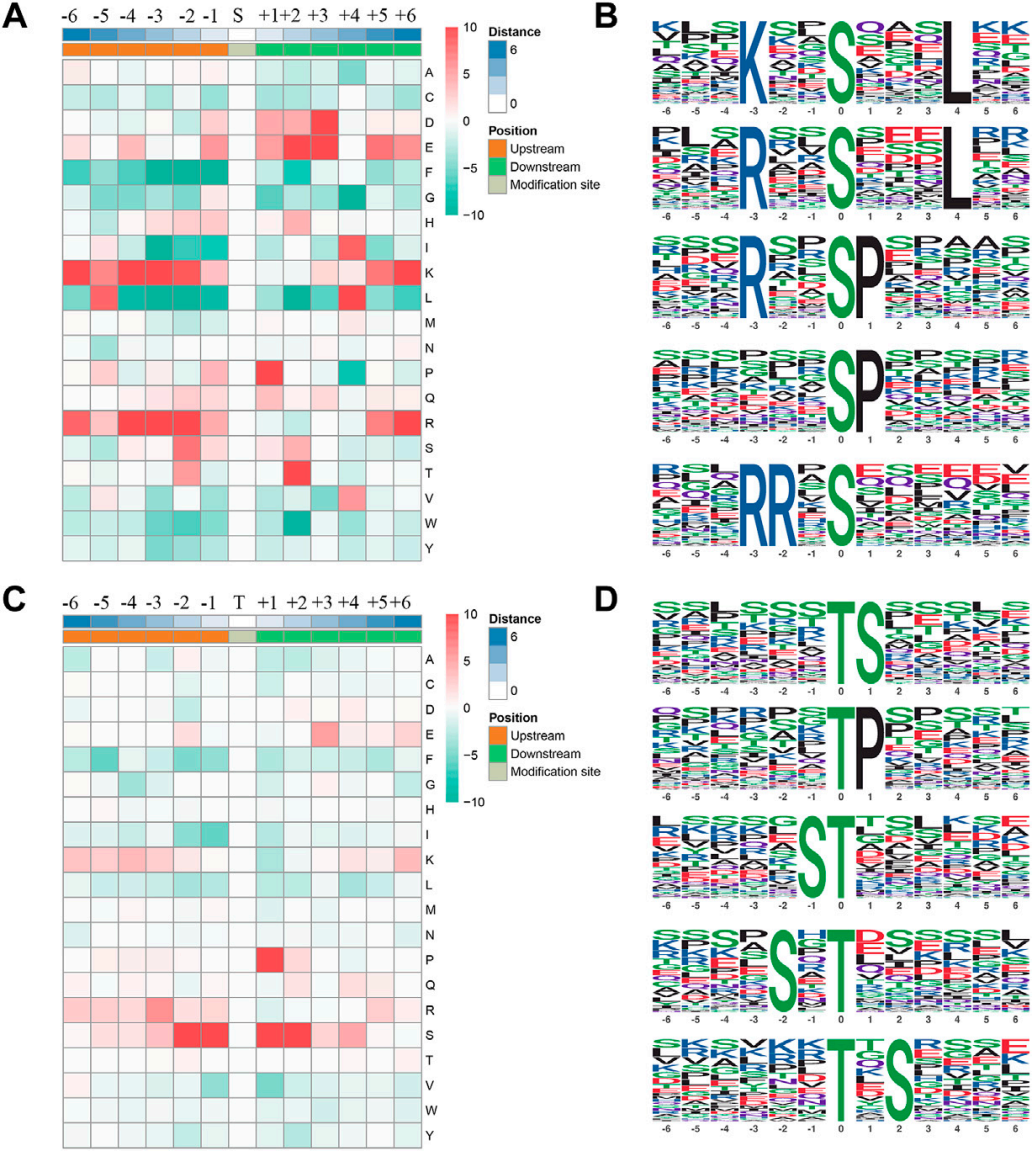
Phosphorylation site motifs. **(A,C)** The statistics of sequence probability in 6 phosphorylation site motifs with the prominent distribution of amino acid around serine and threonine phosphorylation sites. **(B,D)** Top five serine and threonine phosphorylation motifs.

We extracted a 13 amino acid sequences centered on phosphorylation sites and obtained 4,084 distinct sequences, including 3,531 phosphoserines and phosphothreonines (Supplementary Table S2). In addition, by making statistics of the law of amino acid sequences on both sides of the phosphorylation site, the regular trend of amino acid sequences in the region where phosphorylation sites occur was calculated (Figures 4A,C). In such analysis, the sequence characteristics of modification sites could be found, thus determining the modification-related enzymes. The motif-x was used to perform enrichment analysis of the identified data, and a total of 19 serine and 5 threonine sequences were enriched (Figures 4B,D; Supplementary Table S2). Some kinases corresponding to motif sequences were found through literature retrieval and database search. These sequences might be the recognition sites for kinases. Among the proteins phosphorylated by serine, the most common motifs were “KxxSxxxL”, “RxxSxxxL”, “RxxSP”, “SP”, and “RRxS”. Among proteins phosphorylated by threonine, the most common motifs are “TS”, “TP”, and “ST”.

### Functional characteristics and subcellular localization of phosphorylated proteins in yak sperm

**FIGURE 5:**
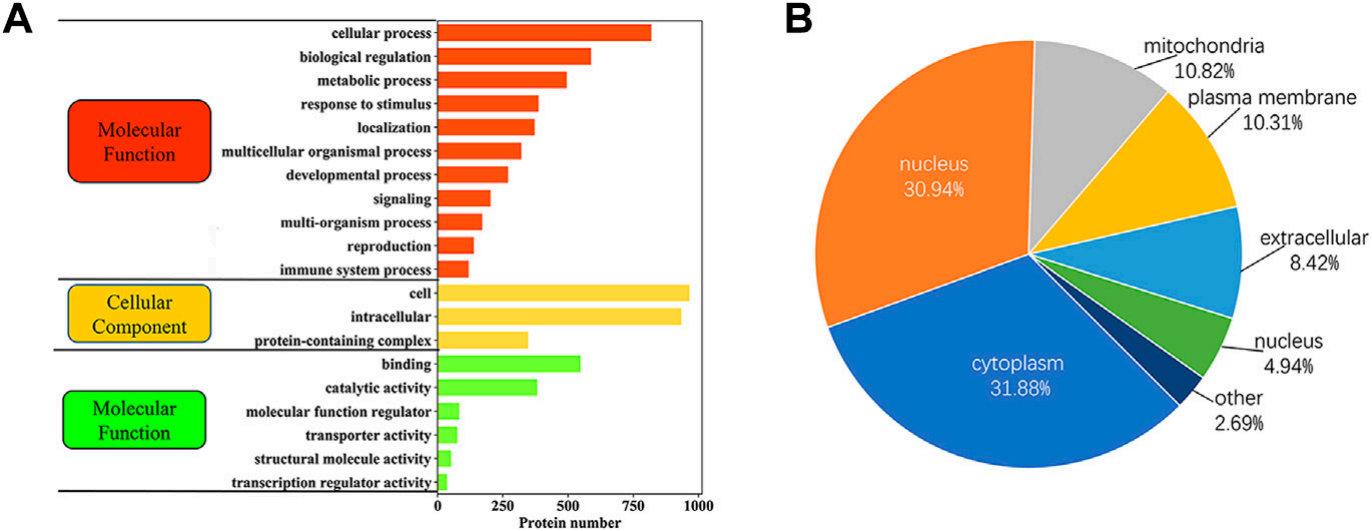
The functional classification of phosphorylated proteins in yak sperm. **(A)** GO functional enrichment of phosphorylated proteins. **(B)** Subcellular localization of phosphorylated proteins.

In order to better understand the potential role of phosphorylated proteins in yak sperm, GO analysis of biological processes (BPs), molecular functions (MFs) and cellular components (CCs) were performed. The results showed as follows: in BPs, most phosphorylated proteins were involved in cellular processes, biological regulation, metabolic processes and reproduction; the most common MFs were protein binding, catalytic activity and molecular function modulation; in CCs, most phosphorylated proteins were involved in cell and protein complexes (Figure 5A; Supplementary Table S3). Besides, several phosphorylated proteins were involved in reproduction. Specifically, proteins involved in reproductive pathway included CABYR, PARK7, AKAP3, and AKAP4. These proteins were related to sperm capacitation and AR during sperm maturation. Besides, subcellular localization showed that phosphorylated proteins were mainly located in the cytoplasm, nucleus, mitochondria, plasma membrane and outside the cells (Figure 5B; Supplementary Table S4).

### The difference of phosphorylated proteins between the different states in yak sperm

**FIGURE 6:**
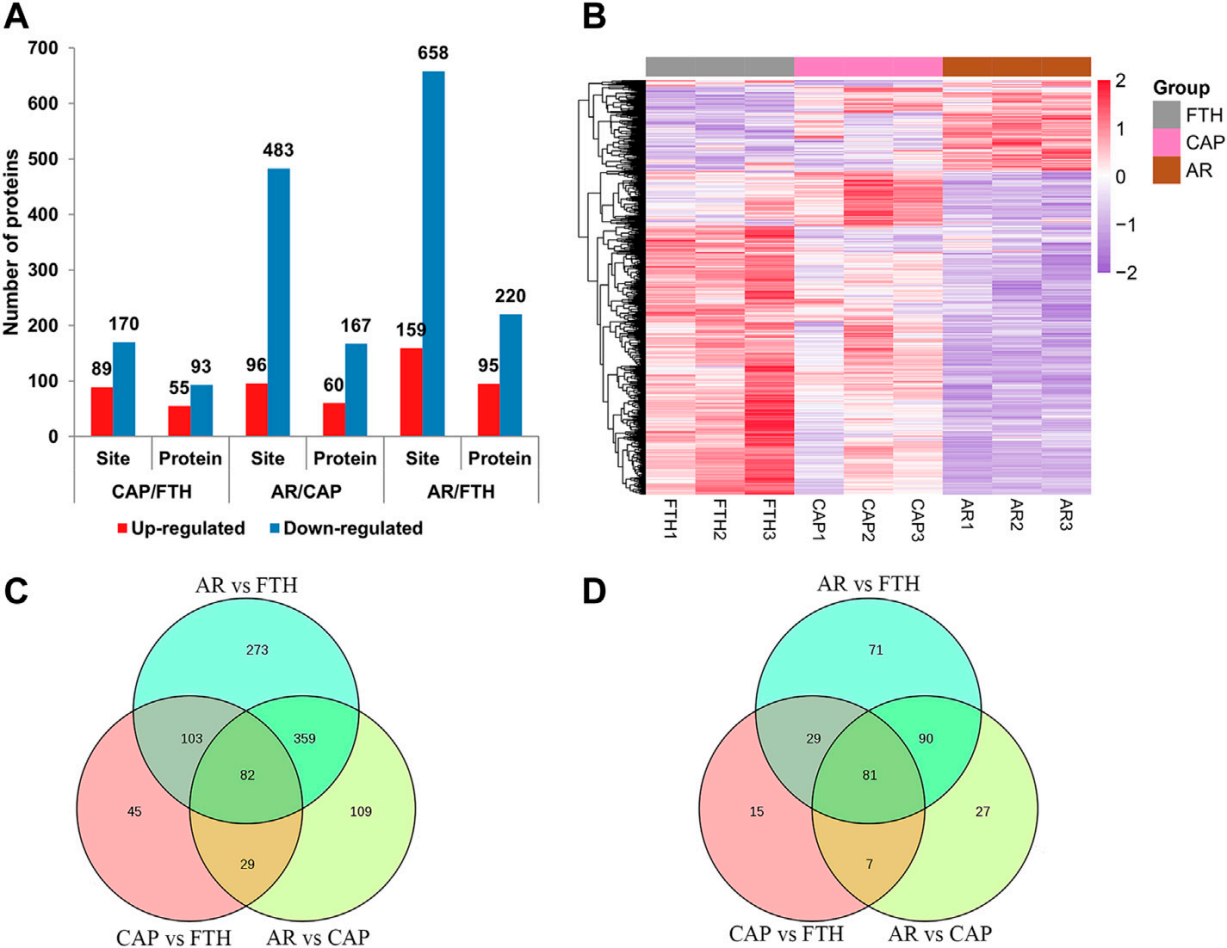
The Identification of differentially expressed proteins under three states of yak sperm (FTH, CAP, and AR). **(A)** The number of differentially expressed phosphorylation sites and their corresponding proteins. **(B)** The cluster analysis of the DEPPs in yak sperm. **(C,D)** The differentially expressed phosphorylation sites and their corresponding proteins in each group were displayed by Venn diagram.

The obtained phosphorylation data were normalized to eliminate the impact of baseline phosphorylation levels. Differentially expressed phosphorylated proteins (DEPPs) were identified by filtering samples for an average cut-off change of 1.2-fold with a *p*-value < 0.05. Briefly, in CAP vs. FTH, 55 proteins and 89 sites were up-regulated, and 93 proteins and 170 sites were down-regulated in total; in AR vs. CAP, 60 proteins and 96 sites were up-regulated, and 167 proteins and 483 sites were down-regulated in total; in AR vs. FTH, 95 proteins and 159 sites were up-regulated, and 220 proteins and 658 sites were down-regulated in total (Figure 6A; Supplementary Table S5). Hierarchical cluster analysis was also conducted for DEPPs, the result of which were illustrated by a heat map (Figure 6B), this demonstrated changes in phosphoprotetomics of yak sperm during capacitation and AR process. The number of shared proteins among the three groups and DEPPs are showed in Figures 6C,D.

### Functional analyses of identified differentially expressed phosphorylated proteins

**FIGURE 7:**
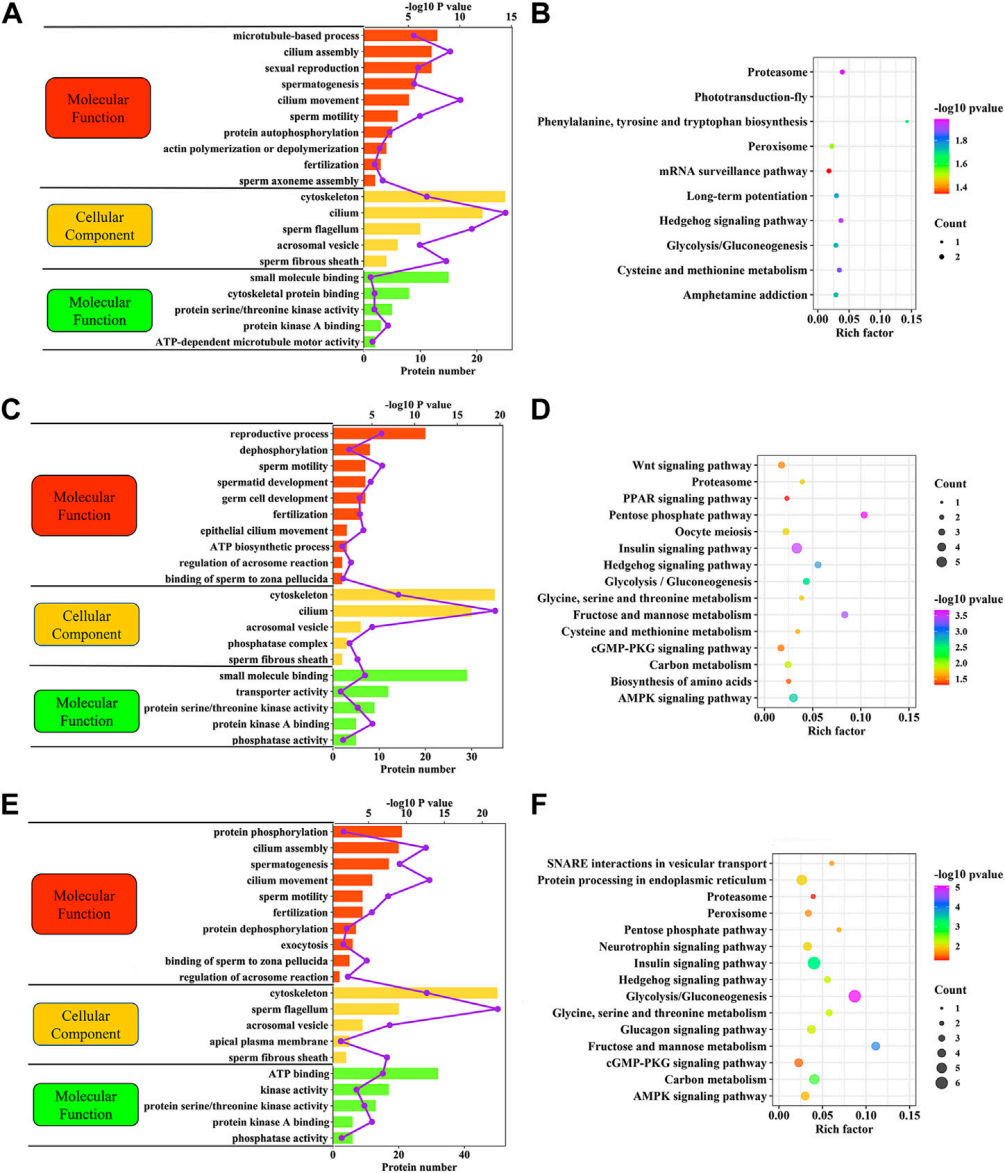
Functional enrichment analyses of DEPPs in yak sperm under capacitation and AR. **(A)** GO term enrichment analysis for CAP vs. FTH sperm comparison. **(B)** KEGG pathway enrichment analysis for CAP vs. FTH sperm comparison. **(C)** GO term enrichment analysis for AR vs. CAP sperm comparison. **(D)** KEGG pathway enrichment analysis for AR vs. CAP sperm comparison. **(E)** GO term enrichment analysis for AR vs. FTH sperm comparison. **(F)** KEGG pathway enrichment analysis for AR vs. FTH sperm comparison.

The potential biological roles of the DEPPs identified were identified using multiple bioinformatics pipelines. GO analyses of DEPPs for the CAP vs. FTH sperm comparison revealed phosphorylated proteins enriched for BPs including cilium movement, sperm motility, spermatogenesis and the fertilization, and CCs including cilium, sperm fibrous sheath and cytoskeleton, as well as MFs including small molecular binding and protein kinase A binding (Figure 7A; Supplementary Table S6). GO analyses of DEPPs for the AR vs. CAP comparison revealed phosphoproteins enriched for BPs including sperm motility, reproductive process, fertilization and dephosphorylation, and CCs including the cilium, cytoskeleton and acrosomal vesicle, as well as MFs including protein kinase A binding, small molecule binding and protein serine/threonine kinase activity (Figure 7C; Supplementary Table S6). GO analyses of DEPPs for AR vs. FTH sperm comparison revealed phosphorylated proteins enriched in BPs including cilium movement, spermatogenesis, sperm motility and fertilization, and CCs including the sperm flagellum, cytoskeleton and acrosomal vesicle, as well as MFs including ATP binding and kinase activity and phosphatase activity (Figure 7E; Supplementary Table S6).

**FIGURE 8:**
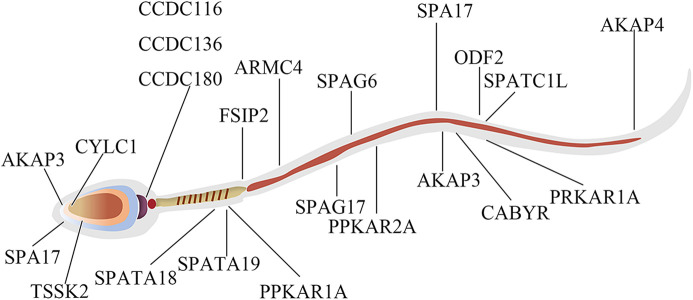
The protein localization map of DEPPs during capacitation and acrosome reaction in yak sperm.

These GO term enrichment data dramatically showed that capacitation and AR was interrelated with changes in the phosphorylation levels of different sperm-specific proteins primarily involved in sperm fertility regulation (Figure 8). Among them, some DEPPs such as AKAP3, AKAP4, ODF2, FSIP2, PRKAR1A, and PRKAR2A related to sperm motility, capacitation and AR, the proteins of the coiled-coil domain-containing protein family (CCDC116, CCDC136, and CCDC180), SPA17 and CABYR were found. These results suggested that regulation of sperm capacitation and AR is tightly coupled with the opposing action of cellular kinases and phosphatases.

As with GO term enrichment, KEGG pathway enrichment of these DEPPs identified in yak sperm differed in between-group comparisons. DEPPs for the CAP vs. FTH sperm comparison were significantly enriched in the metabolism, proteasome and hedgehog signaling pathways (Figure 7B; Supplementary Table S7). DEPPs for the AR vs. CAP sperm comparison were significantly enriched in the metabolism, AMPK and cGMP-PKG signaling pathways (Figure 7D, Supplementary Table S7). DEPPs for the AR vs. FTH sperm comparison were significantly enriched in the metabolism, hedgehog signaling pathway and proteasome (Figure 7F, Supplementary Table S7).

### Protein kinase and phosphatase proteins related to capacitation and acrosome reaction in yak sperm

Protein kinase and phosphatase protein are the two most important antagonistic components involved in protein phosphorylation (Uhrig et al., 2013). In this study, 7 and 3 DEPPs were identified as protein kinases and phosphatases in CAP and FTH sperm comparison, respectively; 13 and 6 DEPPs were identified as protein kinases and phosphatases in AR vs. CAP sperm comparison, respectively; 20 and 7 DEPPs were identified as protein kinases and phosphatases in AR and FTH sperm comparison, respectively (Supplementary Table S8). Interestingly, these protein kinases and phosphatases were characterized by multiple phosphorylation sites. For example, AKAP3 was significantly upregulated at 6 phosphorylation sites and significantly downregulated at 5 phosphorylation sites in three different comparison groups, indicating that it is a potential component of the signaling cascade of sperm maturation. Additionally, the phosphorylation levels of other enzymes such as ACE and GAPDHS in CAP vs. FTH sperm comparison changed significantly; the phosphorylation levels of ALDOA, PSMD4, and ATP1A4 in AR vs. CAP sperm comparison changed significantly (Supplementary Table S8). Some of these enzymes have been showed to be widely involved in sperm capacitation and AR (Rajamanickam et al., 2017; Zigo et al., 2018).

### Characteristics of different phosphorylation states of the same protein in different treatment groups

In this study, 64 DEPPs showed special phosphorylation characteristics among the three comparison groups (Supplementary Table S9). For example, FSIP2 was significantly downregulated at 41 different serine phosphorylation sites and upregulated at 9 ones; it was significantly down-regulated at 5 threonine phosphorylation sites and one up-regulated at The25. CABYR was significantly downregulated at 15 different serine phosphorylation sites, and 2 threonine phosphorylation sites (Supplementary Table S9). The results suggested that the changes in these specific phosphorylation signatures of DEPPs play a positive role in sperm capacitation and AR.

### Protein-protein interaction network of phosphorylated proteins

**FIGURE 9:**
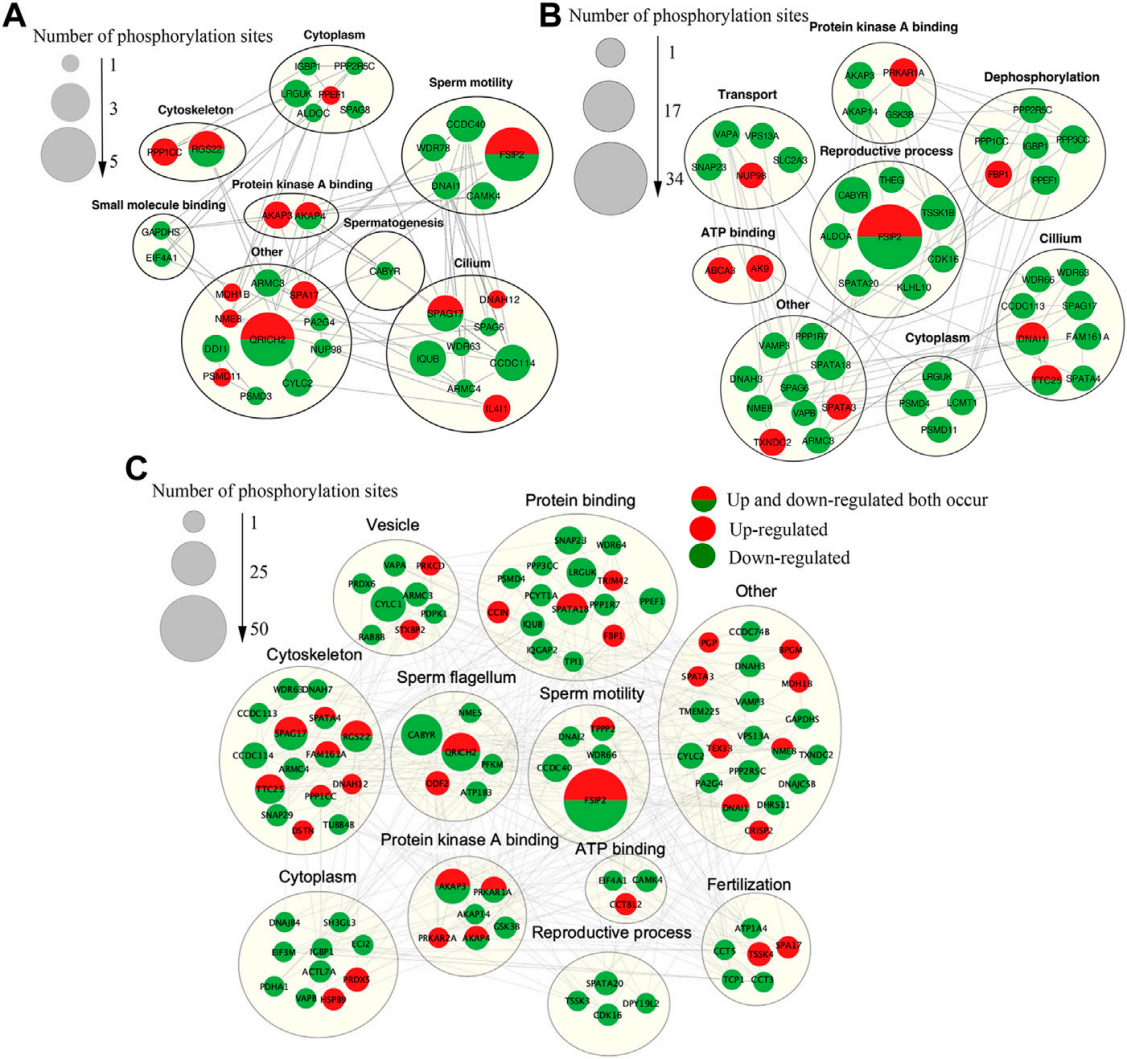
The Interaction network of phosphorylated proteins. **(A)**The PPI network of DEPPs between CAP and FTH sperm. **(B)**The PPI network of DEPPs between AR and CAP sperm. **(C)**The PPI network of DEPPs between AR and FTH sperm. In the constructed network, the number of phosphorylation sites are represented as nodes. While interactions among proteins are represented as edges for proteins with >2 connections. Significantly upregulated and downregulated phosphorylation sites are indicated in red and green, respectively. Red (half) and green (half) showed that upregulated and downregulated phosphorylation sites both occurred. Identified phosphorylated proteins were grouped based on known biological functions.

In order to better understand the biological function of DEPPs in capacitation and AR of yak sperm, a PPI network of identified differentially phosphorylated proteins was assembled based on the STRING database. PPI showed that the main interacting phosphorylated proteins in the three comparison groups were AKAP3, AKAP4, CABYR, SPA17, PPKAR1A, and PPKAR2A, which interacted during sperm capacitation and AR, and also were all related to PKA (Lea et al., 2004; Burton et al., 2006; Liu et al., 2011; Baro Graf et al., 2020). At first glance, the network was organized around several strongly connected sub-networks, the majority of which were directly associated with protein kinase A binding, sperm motility, reproductive process cytoskeleton and sperm flagella, which was related to sperm capacitation and AR (Figures 9A–C). Interestingly, in this study, CABYR was found to be down-regulated in all phosphorylation sites during AR, which occurred on only serine/threonine residues, not tyrosine residues (Figures 9B,C; Supplementary Table S2).

## Discussion

It is worth noting that since sperm are both transcriptionally and translationally silent, their function is highly dependent on the existence of PTMs of the protein complement (Dun et al., 2012; Xu et al., 2021). It is necessary for the regulation of sperm function that phosphorylation as the most important PTM can rapidly control the activity of signaling and regulatory proteins (Parte et al., 2012; Dacheux and Dacheux, 2014). The regulation of protein phosphorylation during capacitation and AR is crucial to sperm function, and moreover, increased levels of tyrosine phosphorylation are considered a marker of capacitation (Morgan et al., 2008; Kwon et al., 2014). In order to understand the overall regulation of phosphorylation during sperm capacitation, some studies have used phosphoproteomics techniques to quantify the changes in phosphorylation sites levels in mice (Platt et al., 2009; Chung et al., 2014; Syifa et al., 2020), rats (Baker et al., 2010)and humans (Wang et al., 2015). Furthermore, to the best of our knowledge, AR has not yet been studied from the perspective of phosphorylated proteomics. In order to better understand the regulation of phosphorylation during yak sperm capacitation and AR, the TMT labeling method of combining IMAC enrichment and LC-MS/MS was adopted to quantify the phosphorylation sites of FTH, CAP and AR sperm in yak. The present study identified 5,509 phosphorylation sites on 1,377 proteins in yak sperm, which represents the first study of bovine sperm phosphorylated proteins and their sites so far. A recent study on human sperm reported that a total of 3,527 phosphorylation sites were identified on 1,322 proteins, of which the five phosphorylated proteins with the most modification sites were FSIP2 (145 sites), AKAP4 (102 sites), AKAP3 (71 sites), CABYR (45 sites), and ODF2 (37 sites) (Urizar-Arenaza et al., 2019), while the five phosphorylated proteins with the most phosphorylation sites in yak sperm were FSIP2 (220 sites), AKAP4 (115 sites), AKAP3 (78 sites), ALMS1(62 sites), and CDC136 (58 sites). Compared with the number of phosphorylated proteins identified in the human sperm proteome, our coverage of the higher-level phosphorylated proteome and its sites in yak sperm helps to ensure reliable downstream analysis of the mechanism of controlling yak sperm capacitation and AR induction. Additionally, the similarity between phosphorylated proteins in yak and humans can also provide theoretical support for the future study of sperm maturation mechanisms in humans and mammals.

Motif analysis of regulatory phosphorylation sites identified by phosphoproteomics data was used to predict protein kinases in response to phosphorylation. It has been reported that “RRxS” is the corresponding motif of PKA, PKB, PKC and PKG, and “SP” is the corresponding motif of MAPK, PKB, PKG and many other kinases (Wang et al., 2019). The two motifs “RxxSP” and “TP” can be recognized by PKA, MAPK and CDK (Wang et al., 2019; Zhang et al., 2021). It is well-known that PKA plays a central role in regulating mammalian sperm capacitation and AR (Baro Graf et al., 2020), and MAPK is also another kinase that regulates sperm capacitation and AR (Mahajan et al., 2022). The phosphoproteomics results in this study showed that some key proteins involved in reproduction, such as ODF2, PPKAR1A, CABYR, AKAP3, AKAP4, and PARK7 were phosphorylated on the motif of PKG, PKA, AKT, PKC, and AMPK, respectively, indicating that these proteins are potential targets for response kinases (Huang et al., 2018; Xu et al., 2021). Considering the identified phosphorylation motif, combined with the specific content of known serine/threonine/tyrosine kinase phosphorylation sites, some important target proteins of upstream kinases involved in sperm capacitation and AR-related signal pathways could be predicted.

Sufficient evidence has shown that protein phosphorylation, especially on Y residues, is the most important event during capacitation (Matamoros-Volante et al., 2018). During human sperm capacitation, the upregulation of AKAP3 and AKAP4 tyrosine phosphorylation levels has been confirmed, and 6 and 2 tyrosine phosphorylation-specific sites have been identified, respectively (Ficarro et al., 2003; Wang et al., 2015). Interestingly, the phosphopeptides identified of SPA17 levels at Y69 sites were found to be significantly upregulated during sperm capacitation in yak. SPA17 is mainly localized in the acrosomal region of the sperm head and the fibrous sheath of the sperm tail, and also localized to a certain extent in the cytoplasm and considered to be a zona pellucida interacting protein (Frayne and Hall, 2002; Lea et al., 2004; Chiriva-Internati et al., 2009; Xu et al., 2021). And it may be involved in the sperm motility and capacitation, AR and fertilization process of the sperm (Chiriva-Internati et al., 2009; Chiriva-Internati, 2011). SPA17 is a three-domain protein and the highly conserved N-terminal domain contains a motif PRKAR2A very similar to the N-terminal sequence of cAMP-dependent PKA, with the presence within this region of an AKAPs-binding motif, suggesting that SPA17 plays an important role in the interaction of AKAPs located in sperm flagella (Frayne and Hall, 2002; Chiriva-Internati et al., 2009). As is well-known, direct activation of PKA by cAMP during sperm capacitation is the key to the occurrence of tyrosine phosphorylation, and the specificity and function of PKA in cells are attributed to its localization through anchoring protein AKAPs in response to cAMP signaling; AKAPs participate in PKA dependent protein tyrosine phosphorylation, and tyrosine phosphorylation levels are both increased in their two members AKAP3 and AKAP4 (Coghlan et al., 1995; Chiriva-Internati et al., 2009; Skroblin et al., 2010; Morielli and O'Flaherty, 2015; Urizar-Arenaza et al., 2019). In turn, AKAPs are also regulated by SPA17, so the interaction between AKAPs and SPA17 causes increased tyrosine phosphorylation during sperm capacitation (Fiedler et al., 2013). Previous studies have shown that cAMP-mediated tyrosine phosphorylation of the sperm tail is critical for sperm capacitation and the occurrence of hyperactivated motility (Aitken and Nixon, 2013). Studies have reported that SPA17 is mainly located in the sperm head and tail, and thus it was speculated by us that SPA17 in the sperm head may also undergo tyrosine phosphorylation during capacitation, which is consistent with the results of recent studies that low levels of tyrosine phosphorylation can also occur in the sperm head (Ruiz-Diaz et al., 2020). Additionally, annotation of the PPI network of proteins with DEPPs during sperm capacitation showed complex relationships between SPA17 and CABYR and between AKAP3 and AKAP4, respectively (Figure 9A). These proteins have been showed to play a crucial role in sperm capacitation (Li et al., 2010; Vizel et al., 2015; Rahamim Ben-Navi et al., 2016). Therefore, this study demonstrated that SPA17 may be a new functional protein in addition to AKAPs family proteins during sperm capacitation.

Although the identification of relatively few tyrosine phosphorylation sites in yak sperm represents a departure from widely accepted models of human sperm capacitation, the results of this study were closer to the situation in somatic cells, in which the estimated proportion of the occurrence of serine, threonine and tyrosine amino acids in phosphorylation is 1000:100:1 (Raggiaschi et al., 2005). In the study of these inconsistent results, it is worth noting that serine/threonine phosphorylated proteins identified by us were instead regulated by tyrosine phosphorylation in human and mouse sperm, thereby increasing the possibility of lineage specific expansion of tyrosine kinase action (Wang et al., 2015; Nixon et al., 2019). Additionally, PKA can also stimulate sperm capacitation and AR through serine/threonine phosphorylation (Leclerc et al., 1996; Kwon et al., 2014), which further demonstrated that serine/threonine phosphorylation also plays a crucial role besides tyrosine phosphorylation during sperm maturation. However, there have been few studies of the proteins with phosphorylated serine and threonine residues during sperm capacitation. In this study, the proteasome signaling pathway was significantly enriched in yak sperm capacitation. Previous studies have reported that proteasome can regulate sperm capacitation by activating PKA activity (Zapata-Carmona et al., 2021), in which proteasome can be phosphorylated by PKA (Kong et al., 2009; Zigo et al., 2018), and that when the proteasome is activated by PKA, it can directly or indirectly regulate the substrate protein phosphorylation of serine and threonine residues through a feedback loop (Kong et al., 2009). PSMD11 is a component of the lid of the 19S RP and plays an important role in regulating 26S proteasome assembly and activity (Vilchez et al., 2012; Lokireddy et al., 2015). In this study, PSMD11 at Ser14 levels was significantly up-regulated during sperm capacitation, indicating that PSMD11 may regulate sperm capacitation by enhancing proteasome activity through increased phosphorylation. The phosphorylated peptide fragments identified in the Ser14 site of PSMD11 in yak contain a specific motif “RxxS”, which is the corresponding motif of PKA (Lokireddy et al., 2015). Therefore, this further demonstrates that PSMD11 phosphorylation may activate cAMP/PKA signaling pathways, which plays a key role during yak sperm capacitation. In highly polarized sperm cells, various compartmentalized functions are regulated by PKA signals. In particular, sperm capacitation is associated with the activation of phosphorylation cascades in which PKA, a serine/threonine kinase, is located upstream of the increase in tyrosine phosphorylation of a few proteins (Visconti et al., 2002). Notably, in this study, Ser735 levels in AKAP4, and The212 and The189 levels in AKAP3 were significantly upregulated during capacitation. Recently, it has been reported that the phosphopeptide identified at the Ser136 site in porcine AKAP4 can enhance the binding effect of AKAP4 to PRKAR2A (Miki and Eddy, 1999; Xu et al., 2021), thus causing the activation of AKAP4, which can link the upstream cAMP/PKA signal pathways to regulate sperm capacitation (Martinez-Heredia et al., 2006). This further demonstrated that serine/threonine modification on AKAPs also plays a crucial role in sperm capacitation.

Although some phosphoproteomics reports have focused on capacitation, this is the first time that a phosphoproteomics strategy has been applied to study the changes at phosphorylated protein sites after AR. The AR initiation required calcium influx. Physiologically, calcium increase is triggered specifically by the progesterone or zona pellucida proteins (Gramajo-Buhler et al., 2012), but the calcium ionophore A23187 treatment serves as an effective surrogate for this calcium influx in this study, because it could lead to an equivalent loss of acrosome content (Castillo et al., 2019). Some studies showed that calcium ionophore A23187 is a stronger inducer of the acrosome reaction that triggers exocytosis in a larger percentage of cells than other more physiological stimuli such as progesterone or zona pellucida proteins (Sosa et al., 2015; Li Y. Y. et al., 2020). During fertilization, sperm need to not only swim to the part of the female reproductive tract where they interact with the egg, but also acquire the ability to fertilize it (Xu et al., 2021). Therefore, sperm motility is the critical factor influencing fertilization. As a matter of fact, several more abundant phosphorylated proteins were already found in acrosome reacted sperm by us, which play an important role in sperm motility, such as FSIP2, DNAI1, ATP1A4, TPPP2, TSK4, AKAP4, and IQCG. Sperm will initiate an increase in Ca^2+^ in this region of cells and the regulation of changes actin network as well as the AR-related Ca^2+^ wave while experiencing AR (Romarowski et al., 2016; Romarowski et al., 2018). In this regard, the impact of cytoskeleton on the propagation and permeability of Ca^2+^ wave has been previously reported (Torregrosa-Hetland et al., 2011). Therefore, the cytoskeleton plays a crucial role in AR and many cytoskeleton-related proteins were found by us in DEPPs, such as ODF2, SPAG17, PPKAR1A, and ALMS1. Additionally, signal transduction pathways regulate sperm capacitation and AR, and the signal transduction cascade that triggers sperm AR depends on the plasma membrane in the head (Abou-haila and Tulsiani, 2009). Through KEGG enrichment analysis, some phosphorylation regulatory proteins were found to be involved in AMPK and cGMP-PKG signaling pathways in signal transduction. AMPK is a highly conserved serine/threonine kinase, which can serve as a key cellular sensor to enhance ATP-producing pathways and inhibit ATP-consuming metabolic ones (Hardie et al., 2006; Carling et al., 2012). The cellular energy level is a crucial determinant of sperm motility, AR induction and subsequent fertilization (Nguyen et al., 2014). Furthermore, AMPK is also regulated by upstream PKA (Nguyen et al., 2019). The activation of AMPK in sperm is beneficial for enhanced motility, AR induction and successful oocyte fertilization (Hurtado de Llera et al., 2015; Nguyen, 2017).

As mentioned earlier, many phosphorylated proteins, protein kinases and phosphatases contained in mammalian sperm participate in sperm motility, capacitation and AR (Garbers et al., 1980; Tash and Means, 1983). Among them, activated PKA catalyzes the phosphorylation of Ser/Thr residues of other proteins, thus further activating other protein kinases, which is the key to AR (Da Ros et al., 2004). PRKAR1A and PRKAR2A identified in these proteins by us are two kinases that regulate cAMP- dependent protein kinase A. PRKAR1A is mainly expressed in sperm’s outer dense fibers and fibrous sheath, and PRKAR2A in the axonal region of sperm flagella (Fiedler et al., 2008; D’Amours et al., 2018). PRKAR2A contains a phosphorylation site in the inhibitory domain, while the PRKAR1A subunit does not; this may lead to the altered binding affinity for the catalytic subunits, and they both dimerize and bind to AKAPs through their N-terminal domains (Wu et al., 2007). The upregulation of phosphorylation of PRKAR1A (Ser76/82/306) and PRKAR2A (Ser380) was identified in acrosome reacted sperm in this study respectively, indicating that two PKA subunits play different roles during AR process. These two proteins were found to both interact with AKAP3, AKAP4, and CABYR through PPI (Figures 9B,C). The AKAPs protein family is one of the main components of sperm fibrous sheath, which can regulate sperm capacitation and AR by binding to other protein kinases, protein phosphatases, ion channels and small GTP binding proteins as modulators integrating cAMP/PKA and PKC/ERK1/2 signal pathways (Skroblin et al., 2010; Rahamim Ben-Navi et al., 2016). For one thing, the phosphorylation level of AKAP3 (Ser75/132) decreased after AR in this study, suggesting that AKAP3 may be correlated with PKC/ERK1/2) signal pathways downstream of AR. For another, there is evidence for the interaction and coexpression of AKAP3 and CABYR in human sperm (Li et al., 2011; Mallon et al., 2013). CABYR is localized the midpiece of sperm as an essential component of hyperactivation and calcium signaling pathways during capacitation (Urizar-Arenaza et al., 2019). CABYR has self-assembly and putative motifs that bind PRKAR2A and AKAPs (Li et al., 2011), and its dephosphorylation eliminates its ability to bind to calcium (Naaby-Hansen et al., 2002). Surprisingly, it was observed by us that CABYR were all dephosphorylated after AR (Figures 9B,C), and also such dephosphorylation occurred on only serine/threonine residues, not tyrosine residues. To our knowledge, this is the first study in which the dephosphorylation of CABYR on serine/tyrosine residues during AR was demonstrated. The results suggested that the AR involves serine/threonine-Pro phosphatase, which dephosphorylates CABYR, thereby triggering a biological reaction. The dephosphorylation of CABYR on a large number of Ser/Thr Pro residues also indicate that CABYR eliminate binding to calcium during this process. Based on these observations, we hypothesize that the phosphorylated form of CABYR can sequester molecules involved in regulating sperm mobility and exocytosis. Dephosphorylation of CABYR during AR will lead to their functional switch, which changes their affinity with different client proteins, thus inducing hyperactivated motility and AR. Apart from that, previous studies have shown that the role of AKAPs as scaffolds that integrate cAMP/PKA, Rho and calcium signals reinforces the speculation that AKAP3 and CABYR may be linkers between different transduction cascades (Skroblin et al., 2010).

## Conclusion

In this work, we reported the phosphorylation regulatory mechanisms associated with sperm capacitation and AR in yak by phosphoproteomics coupled with bioinformatics analysis. This study revealed some new phosphorylated proteins in cAMP/PKA signal transduction pathways, among which tyrosine phosphorylation levels in SPA17 increased during yak sperm capacitation, which suggested SPA17 may be a new functional protein during sperm capacitation. Moreover, the phosphorylation of PRKAR1A and PRKAR2A, and the dephosphorylation of CABYR occur during yak sperm AR. These new findings contribute to an in-depth understanding of phosphorylated proteins as the primary regulator during sperm maturation. It is believed that the phosphorylated proteome data on Ashidan yak sperm will accelerate further research in the context of male fertility.

## Data Availability

The data presented in the study are deposited in the ProteomeXchange repository with the accession number PXD035751.
