# Supplementary material for: Quantitative phosphoproteomics analyses reveal the regulatory mechanisms related to frozen-thawed sperm capacitation and acrosome reaction in yak (Bos grunniens)
Source: Front Physiol. 2022 Oct 6;13:1013082. doi: 10.3389/fphys.2022.1013082 (PMC9583833; doi:10.3389/fphys.2022.1013082)
Supplement: Supplementary file 1 [file DataSheet1.ZIP › supplementary materials/GO script.pdf]

```
#!/usr/bin/env python
# coding: utf-8

# In[1]:

import pandas as pd
import matplotlib.pyplot as plt
import seaborn as sns
import numpy as np
import os

# In[3]:

from matplotlib import rcParams
rcParams['font.family'] = 'Times new roman'
rcParams['font.size'] = 16
rcParams['font.weight'] = 'bold'
#colors = ['chartreuse1', 'coral1','darkgoldenrod1']
colors = ["#FF501A", "#FFD84C","#A5FF4C"]
sns.set_palette(sns.color_palette(colors))

def plot(df, comparison, outName):
    fig, ax  = plt.subplots(figsize=(8,8), tight_layout=True)
    ty = ax.twiny()
    ax.barh(width = 'Protein number', y = 'Description', color = "color", height = 0.7,  data
= dfsort)
    sns.pointplot(x='logP', y='Description', data = dfsort, s = 10, ax = ty, color = 'blueviolet')
    ty.set_xlabel('-log10 P value', size=16, weight='bold')
    ax.set_xlabel('Protein number', weight = 'bold')
    ax.set_ylabel('CAP vs FTH')
    ax.set_ylabel(comparison, weight='bold')
    plt.savefig('{0}.png'.format(outName), dpi = 500, facecolor='white')
    plt.savefig('{0}.pdf'.format(outName), dpi = 500, facecolor='white')

# In[150]:

compDict = {0:'CAP vs FTH', 1:'AR vs CAP', 2: 'AR vs FTH'}
sheetIDX = 2
comp = compDict[sheetIDX]
outName = comp.replace(' ', '_')
df1 = pd.read_excel('GO.xlsx', sheet_name=sheetIDX)
df1 = df1.dropna(axis = 1)
df1['logP'] = np.log10(df1['pvalue']) * -1
colors = ["#FF501A", "#FFD84C","#A5FF4C"]
```

```
cDict = dict(zip(list(df1['GO Term'].unique()),colors ))
print(cDict)
dfsort = pd.DataFrame()
for cat, grp in df1.groupby('GO Term'):
    print(cat)
    grp = grp.sort_values(by = 'Protein number', ascending=False)
    grp['color'] = cDict[cat]
    dfsort = dfsort.append(grp)
dfsort

# In[151]:

plot(dfsort, comp, outName)

# In[5]:

from matplotlib import rcParams
rcParams['font.family'] = 'Times new roman'
rcParams['font.size'] = 16
rcParams['font.weight'] = 'bold'
#colors = ['chartreuse1', 'coral1','darkgoldenrod1']
colors = ["#FF501A", "#FFD84C","#A5FF4C"]
sns.set_palette(sns.color_palette(colors))

def plot(df, comparison, outName):
    fig, ax    = plt.subplots(figsize=(8,8), tight_layout=True)
    ty = ax.twiny()
    ax.barh(width = 'Protein number', y = 'Description', color = "color", height = 0.7,    data
= dfsort)
    sns.pointplot(x='logP', y='Description', data = dfsort, s = 10, ax = ty, color = 'blueviolet')
    ty.set_xlabel('-log10 P value', size=16, weight='bold')
    ax.set_xlabel('Protein number', weight = 'bold')
    ax.set_ylabel('CAP vs FTH')
    ax.set_ylabel(comparison, weight='bold')
    plt.savefig('{0}.png'.format(outName), dpi = 500, facecolor='white')
    plt.savefig('{0}.pdf'.format(outName), dpi = 500, facecolor='white')

# In[2]:

df1 = pd.read_excel('GO_WO_P.xlsx', sheet_name=0)
df1 = df1.dropna(axis = 1)
df1
```

```
# In[4]:

colors = ["#FF501A", "#FFD84C","#A5FF4C"]
cDict = dict(zip(list(df1['GO term'].unique()),colors ))
print(cDict)

# In[17]:

dfsort = pd.DataFrame()
for cat, grp in df1.groupby('GO term'):
        print(cat)
        grp = grp.sort_values(by = 'Protein number', ascending=True)
        grp['color'] = cDict[cat]
        dfsort = dfsort.append(grp)
dfsort

# In[18]:

l1 = list(cDict.keys())
l1.reverse()
l1

# In[22]:

comparison = 'Phosphorylated proteins'
outName = 'Phosphorylated_proteins_go'

from matplotlib import rcParams
rcParams['font.family'] = 'Times new roman'
rcParams['font.size'] = 16
rcParams['font.weight'] = 'bold'

fig, ax    = plt.subplots(figsize=(8,8), tight_layout=True)
ytickLab = list(df1['Description'].values)
ytickLab.reverse()
for cat in l1:
        print(cat)
        dfplot = dfsort.loc[dfsort['GO term'] == cat]
        ax.barh(width = 'Protein number', y = 'Description', color = "color", height = 0.7,    data
= dfplot)

ax.set_ylabel('CAP vs FTH')
ax.set_xlabel('Protein number', weight='bold')
ax.set_ylabel(comparison, weight='bold')
```

```
ax.set_ylim(-0.5,19.5)
#ax.set_yticklabels(ytickLab, weight='bold')
plt.savefig('{0}.png'.format(outName), dpi = 500, facecolor='white')
plt.savefig('{0}.pdf'.format(outName), dpi = 500, facecolor='white')

# In[ ]:
```
