# Supplementary material for: Quantitative phosphoproteomics analyses reveal the regulatory mechanisms related to frozen-thawed sperm capacitation and acrosome reaction in yak (Bos grunniens)
Source: Front Physiol. 2022 Oct 6;13:1013082. doi: 10.3389/fphys.2022.1013082 (PMC9583833; doi:10.3389/fphys.2022.1013082)
Supplement: Supplementary file 1 [file DataSheet1.ZIP › supplementary materials/KEGG script.pdf]

```
######## KEGG bubble plot

library(ggplot2)
library(xlsx)
library(RColorBrewer)
library("scales")
setwd('/Users/M257071/Documents/Guangchao Sun/Graphing/纯度—july')
rm(list=ls())


##### sheet 1 (CAP vs FTH)
fin <- xlsx::read.xlsx(file ='KEGG.xlsx', sheetIndex = 1)
dfkegg1 <- as.data.frame(fin)
dfkegg1$"-log10 pvalue" = log10(dfkegg1$Pvalue)* -1
dfkegg1$Count = as.factor(dfkegg1$Protein.number)
dfkegg1 <-   na.omit(dfkegg1)
attach(dfkegg1)


p1 <-ggplot(data = dfkegg1, aes(x=Pathway, y=Rich.factor, color = `-log10 pvalue`)) +
  geom_point(aes(size=Count)) +
  scale_colour_gradientn(colours=rainbow(5)) +
  scale_size_manual(values = c("1" = 1, "2" = 2)) +
  theme_bw(base_size = 14) +
  labs(subtitle = "CAP vs FTH") +
  ylab("Rich factor")+
  scale_y_continuous(limits = c(0, 0.15), breaks=pretty_breaks(4)) +
  theme(text = element_text(family = "Times New Roman",color = 'black', face='bold')) +
  guides(fill=guide_legend(title="-log10 pvalue")) +
  theme(axis.text.x = element_text(color = "black", size = 14, face = "bold"),
          axis.text.y = element_text(color = "black", size = 14, face = "bold"),
  ) +
  coord_flip()
#theme(panel.grid.major = element_blank(), panel.grid.minor = element_blank())
p1

fout = 'KEGG_plot_cap_vs_fth.png'
fout_pdf = 'KEGG_plot_cap_vs_fth.pdf'
ggsave(p1, filename = fout, dpi = 500,
        width = 8, height = 5, units = "in")

ggsave(p1, filename = fout_pdf, device = cairo_pdf,
        width = 8, height = 5, units = "in")
```

```
##### sheet 2 (AR vs CAP)
fin <- xlsx::read.xlsx(file ='KEGG.xlsx', sheetIndex = 2)
dfkegg2 <- as.data.frame(fin)
dfkegg2$"-log10 pvalue" = log10(dfkegg2$Pvalue)* -1
dfkegg2$Count = as.factor(dfkegg2$Protein.number)
dfkegg2 <-   na.omit(dfkegg2)
attach(dfkegg2)
unique(dfkegg2$Protein.number)
p2 <-ggplot(data = dfkegg2, aes(x=Pathway, y=Rich.factor, color = `-log10 pvalue`)) +
  geom_point(alpha = 0.7, aes(size=Count)) +
  scale_colour_gradientn(colours=rainbow(5)) +
  scale_size_manual(values = c("1" = 1, "2" = 2, "3" = 3, "4" = 4, "5" = 5)) +
  theme_bw(base_size = 14) +
  labs(subtitle = "AR vs CAP") +
  ylab("Rich factor")+
  scale_y_continuous(limits = c(0, 0.15), breaks=pretty_breaks(4)) +
  theme(text = element_text(family = "Times New Roman",color = 'black', face='bold')) +
  theme(axis.text.x = element_text(color = "black", size = 14, face = "bold"),
        axis.text.y = element_text(color = "black", size = 14, face = "bold"),
  ) +
  coord_flip()
p2
fout = 'KEGG_plot_ar_vs_cap.png'
fout_pdf = 'KEGG_plot_ar_vs_cap.pdf'

ggsave(p2, filename = fout, dpi = 500,
       width = 8, height = 5, units = "in")

ggsave(p2, filename = fout_pdf, device=cairo_pdf,
       width = 8, height = 5, units = "in")

##### sheet 3 (AR vs FTH)
fin <- xlsx::read.xlsx(file ='KEGG.xlsx', sheetIndex = 3)
dfkegg3 <- as.data.frame(fin)
dfkegg3$"-log10 pvalue" = log10(dfkegg3$Pvalue)* -1
dfkegg3$Count = as.factor(dfkegg3$Protein.number)
dfkegg3 <-   na.omit(dfkegg3)
attach(dfkegg3)
unique(dfkegg3$Protein.number)
sizeRange <- c(2,12)
p3 <-ggplot(data = dfkegg3, aes(x=Pathway, y=Rich.factor, color = `-log10 pvalue`)) +
  geom_point(alpha = 0.7, aes(size=Count)) +
  scale_colour_gradientn(colours=rainbow(5)) +
  scale_size_manual(values = c("1" = 1, "2" = 2, "3" = 3, "4" = 4, "5" = 5, "6" = 6 )) +
```

```
    theme_bw(base_size = 14) +
    labs(subtitle = "AR vs FTH") +
    ylab("Rich factor")+
    scale_y_continuous(limits = c(0, 0.15), breaks=pretty_breaks(4)) +
    theme(text = element_text(family = "Times New Roman",color = 'black', face='bold')) +
    theme(text = element_text(family = "Times New Roman",color = 'black', face='bold')) +
    theme(axis.text.x = element_text(color = "black", size = 14, face = "bold"),
            axis.text.y = element_text(color = "black", size = 14, face = "bold"),
    ) +
    coord_flip()
p3

fout = 'KEGG_plot_ar_vs_fth.png'
fout_pdf = 'KEGG_plot_ar_vs_fth.pdf'
ggsave(p3, filename = fout, dpi = 500,
            width = 8, height = 5, units = "in")

ggsave(p3, filename = fout_pdf, device=cairo_pdf,
            width = 8, height = 5, units = "in")
```
