# Supplementary figures and images for: Quantitative phosphoproteomics analyses reveal the regulatory mechanisms related to frozen-thawed sperm capacitation and acrosome reaction in yak (Bos grunniens)
Source: Front Physiol. 2022 Oct 6;13:1013082. doi: 10.3389/fphys.2022.1013082 (PMC9583833; doi:10.3389/fphys.2022.1013082)

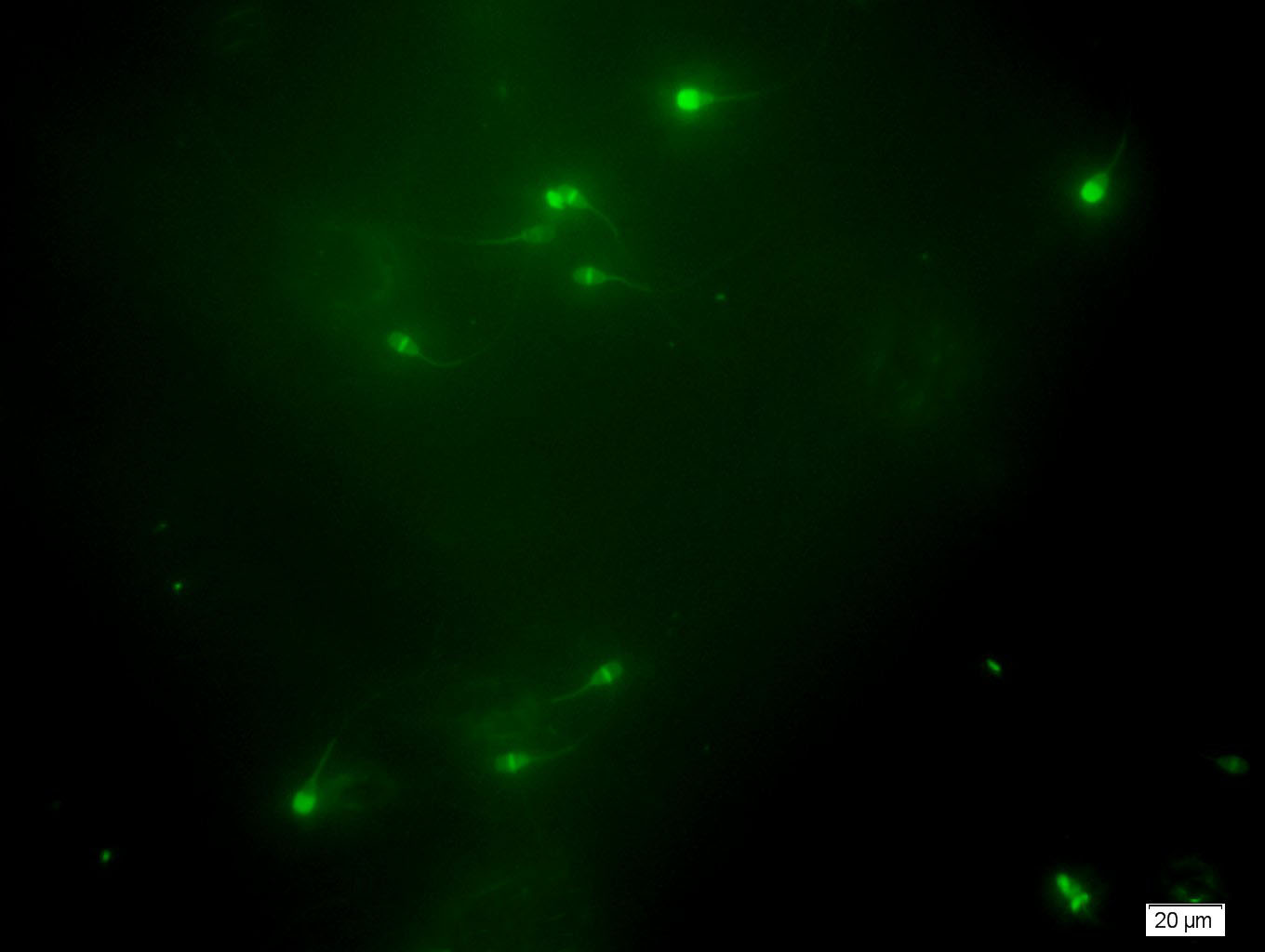

Supplement: Supplementary file 1 [file DataSheet1.ZIP › supplementary materials/AR.jpg]

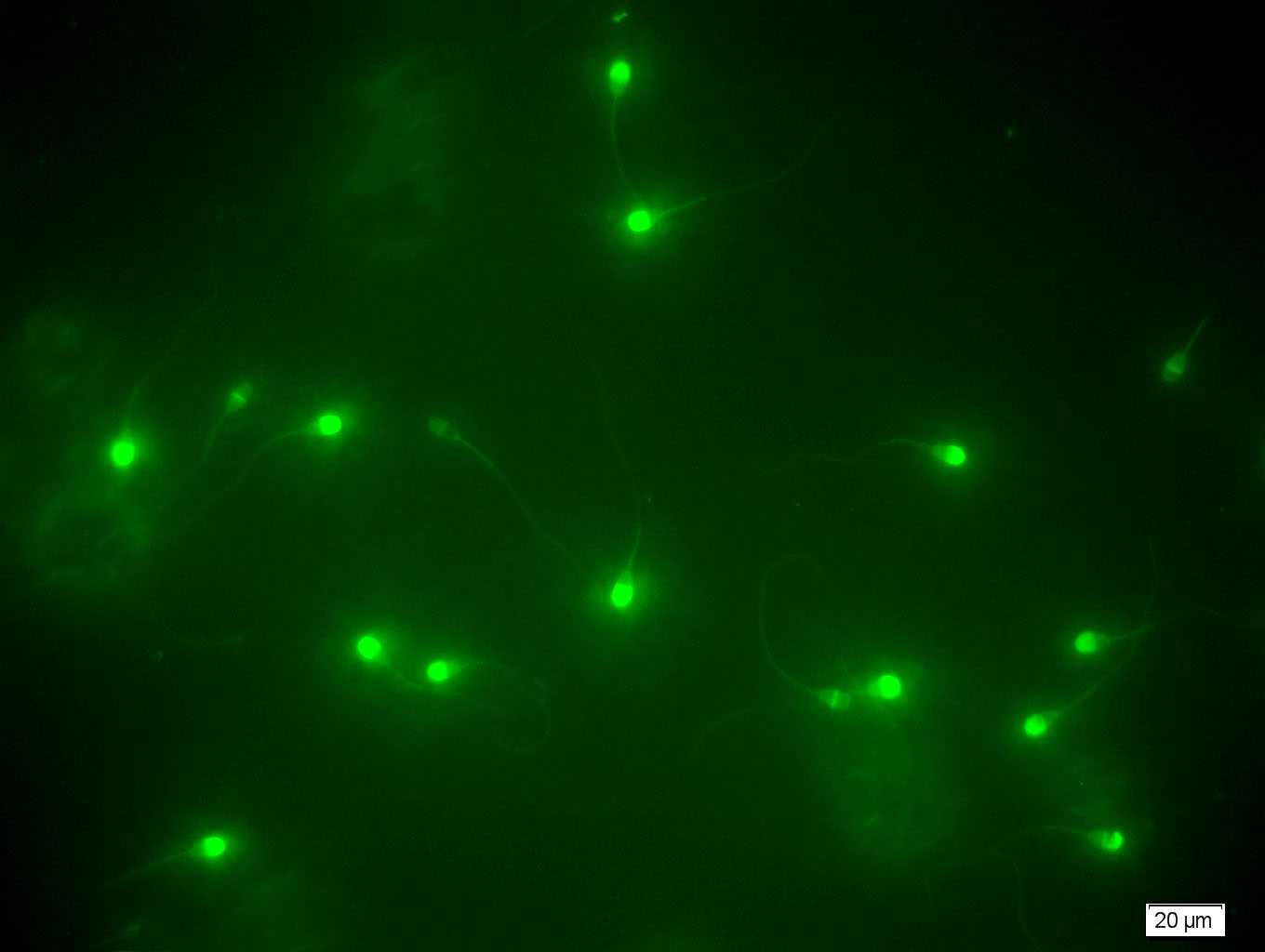

Supplement: Supplementary file 1 [file DataSheet1.ZIP › supplementary materials/CAP.jpg]

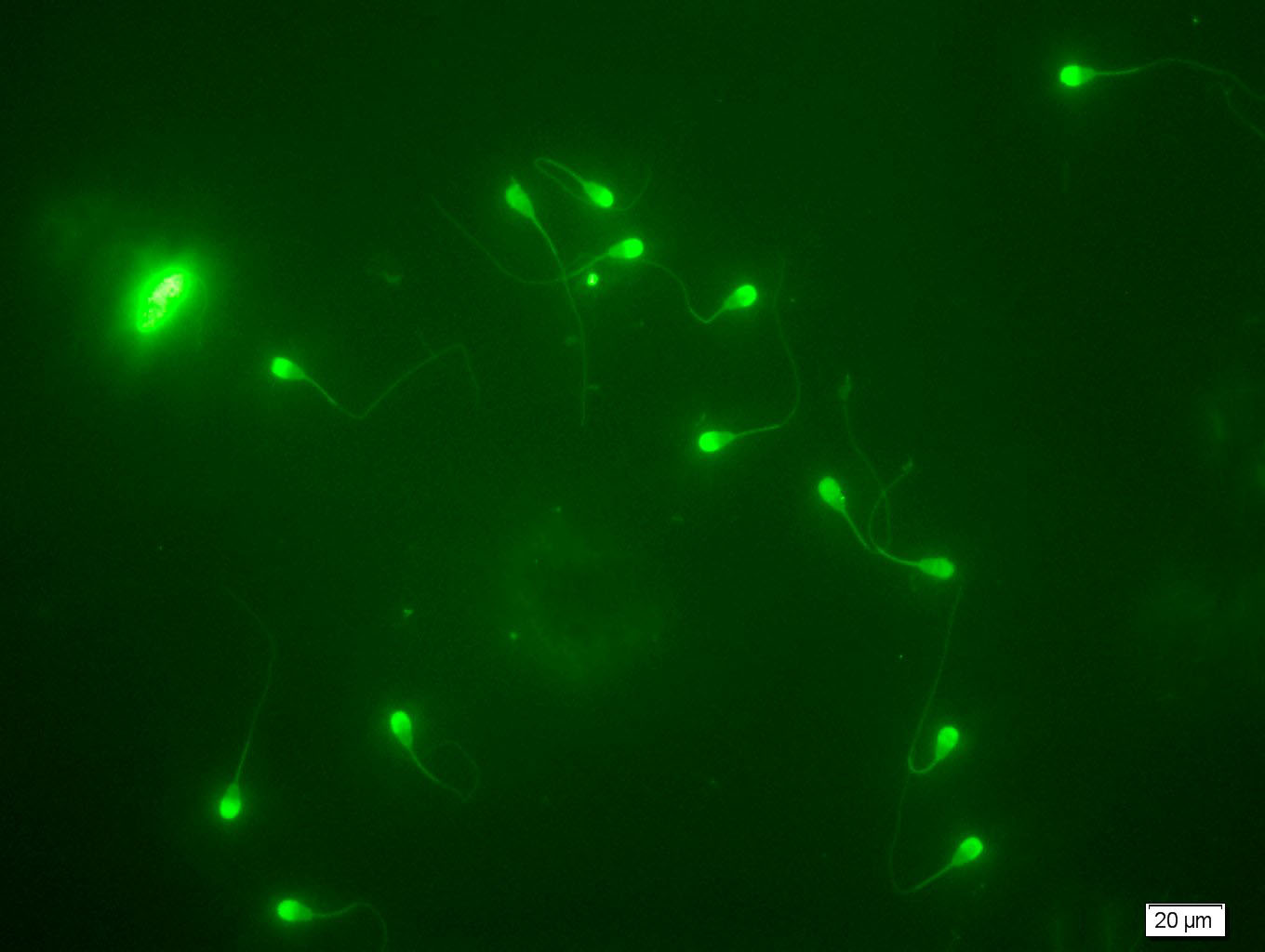

Supplement: Supplementary file 1 [file DataSheet1.ZIP › supplementary materials/DMSO.jpg]

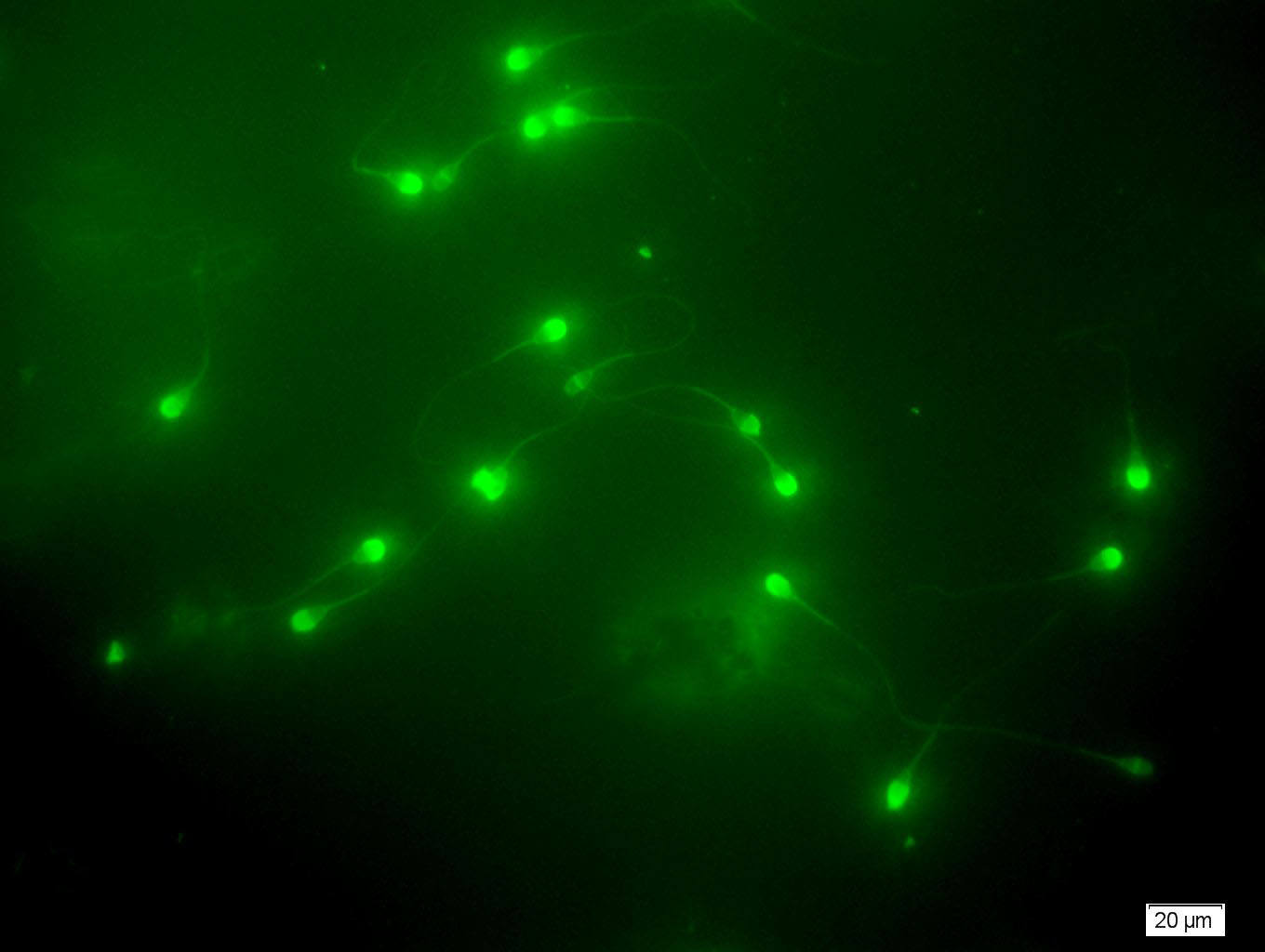

Supplement: Supplementary file 1 [file DataSheet1.ZIP › supplementary materials/FTH.jpg]
